# Twenty-four years lucerne (*Medicago sativa* L.) breeder seed production in India: a retrospective study

**DOI:** 10.3389/fpls.2023.1259967

**Published:** 2023-10-26

**Authors:** Subhash Chand, Ajoy Kumar Roy, Tejveer Singh, Rajiv Kumar Agrawal, Vijay Kumar Yadav, Sanjay Kumar, Devendra Ram Malaviya, Amaresh Chandra, Devendra Kumar Yadava

**Affiliations:** ^1^ ICAR-Indian Grassland and Fodder Research Institute, Jhansi, India; ^2^ ICAR-National Research Centre on Seed Spices, Ajmer, India; ^3^ ICAR-Indian Agricultural Research Institute, New Delhi, India

**Keywords:** breeder seed, lucerne, quality seed, varietal replacement rate, fodder crop

## Abstract

Lucerne (*Medicago sativa* L.) is the second most significant winter leguminous fodder crop after berseem in India. Breeder seed (BS) is the first stage of the seed production chain, as it is the base material for producing foundation and certified seeds. In India, lucerne BS demand has been reduced by 85.58% during the last 24 years (1998–1999 to 2021–2022), declining from 2150 kg to 310 kg. Out of 14 varieties released and notified so far, only nine varieties entered the seed chain since 1998–1999. It shows narrow varietal diversification and, hence, needs robust breeding programs towards enriching genetic variability and varietal development. The present study also highlights the disparity in BS demand and production over the years and puts forth the possible reasons behind the reduction in BS demand and production in the country. Out of the nine varieties, the BS demand of Anand-2 (53.11%) was highest, followed by Type-9 (19.44%) and RL-88 (13.60%). Varietal replacement rate (VRR) was found to be moderate, i.e., 23.67% for the varieties having <5 years old age in the last 3 years (2019–2020 to 2021–2022). It has also been estimated that BS produced (233 kg) during 2021–2022 can cover the approximate area of 6,300 ha at farmers’ fields in 2024–2025 if the seed chain functions 100%, effectively. The present study provides a holistic overview of lucerne BS demand and production, challenges in BS production, and the way forward to develop more varieties and surplus BS production in the country.

## 1 Introduction

Lucerne (*Medicago sativa* L.), also known as alfalfa or *rijika* in Hindi, is an important crop in the temperate agro-climatic regions globally and has the highest productivity of feed protein per unit area (Annicchiarico et al., 2010; 2015). It is an autotetraploid crop with 2n=4x=32 originating in north-western Iran and north-eastern Turkey (Irwin et al., 2001; Wang and Şakiroğlu, 2021). It is mainly preferred for hay production and quality pasture for livestock due to its high protein content (Gaweł, 2008; Babu et al., 2014). Lucerne, as a fodder crop, has better adaptability over the other grasses and legumes due to its nutritional superiority, having a high content of proteins, vitamins, and minerals, high green fodder, and good atmospheric nitrogen fixation (Lamb et al., 2006; Bouton, 2012). However, the genetic gain of forage yield in lucerne is low (0.2%–0.3% per year) as compared to maize [*Zea mays* L., 2%] and white clover [*Trifolium repens* L.; 1%] (Woodfield and Brummer, 2001; Lamb et al., 2006). The genetic gain is influenced by various factors such as autotetraploid nature, perennial growth habit, highly cross-pollinating system, high level of non-additive genetic variance due to gene interaction, and genotype-by-environment interaction (GEI) (Woodfield, 1999; Kapadia, 2019).

In India, lucerne is predominantly cultivated in subtropical and tropical climatic conditions as a major *Rabi* fodder crop and is estimated to cover 1.0 Mha area (Chauhan et al., 2017). *Rabi* is the winter season in India, where crops are sown in October–November and are harvested in April–June. The states having the highest area under cultivation are Gujarat, Rajasthan, Maharashtra, Punjab, Haryana, Madhya Pradesh, Uttar Pradesh, Tamil Nadu, and Karnataka (Roy et al., 2020). In the northern and central states like Uttar Pradesh, Madhya Pradesh, Haryana, and Punjab, it is taken as a multicut winter annual crop during the period October–June, whereas in western states like Gujarat, Rajasthan, and Maharashtra, it is cultivated as a perennial multicut crop with 3 years rotation.

There is a well-organized and carefully defined system of variety release and notification in India. All India Coordinated Research Projects (AICRPs) in different crops arrange multilocation and multiyear testing of all the entries contributed from different public and private sector institutions along with national, zonal, and local checks in coded form (Chand et al., 2020). In annual crops, three cycles of evaluation are followed: initial varietal trial (IVT) and advanced varietal trials (AVT-1, AVT-2) with defined promotion criteria at each stage. However, the perennial crops require 4 years—1 year for crop establishment and 3 years for evaluation under coded form in the same field under multicut system. The data of 3 years are pooled and analyzed. Various agro-morphological parameters, green and dry matter yield, nutritive parameters, tolerance to major biotic stress, etc. are recorded along with seed production potential and agronomic responses such as phosphorus use efficiency, cutting and irrigation schedule. Based on cumulative 3 years’ result, a decision is taken by a duly constituted Varietal Identification Committee based on merit. If identified, it is put before the Central Varietal Release Committee, a statutory body of the Government of India. If approved, it is notified in the gazette for cultivation.

The seed chain in any crop could be sustainable and effective only when breeder seed (BS) production meets the BS demand. BS is the progeny of nucleus seed and is produced by the concerned institutions that have developed the variety like ICAR institutes or State Agricultural Universities (SAUs)/agencies with the help of Project Coordinators (PCs)/Project Directors (PDs) of AICRPs in the different crops (https://seednet.gov.in/). In lucerne, only public sector-bred varieties come into the seed chain system for seed multiplication; however, the varieties developed by the private sector and notified through the Central Variety Release Committee (CVRC) do not proceed into the seed chain and sell their seeds directly to the farmers. In India, BS production is the mandate and responsibility of the ICAR and DAC (Department of Agriculture, Cooperation and Farmers Welfare, Government of India) compiles the BS indents of states, union territories (UTs), public sector units (PSUs), and private seed companies in different crops and provide them to the concerned authorities of ICAR, *viz.*, PCs/PDs for the production of the BS in each crop. The indents are then allocated to the concerned breeder/parent institute for BS production after considering factors like the availability of nucleus seed and other facilities at the center. Monitoring is done by the duly constituted committee that includes the breeder of the variety, the concerned PC/PD or his or her nominee, and one member of the National Seed Corporation (NSC) at regular intervals as per ICAR guidelines (https://seednet.gov.in/). AICRP on Forage Crop and Utilization (AICRP FC&U) plays a vital role in the supervision and coordination of maintenance and production of nucleus and breeder seed and their supply network, thereby indirectly helping in the production of the required quantity of foundation and certified seed in the country. Varietal replacement rate (VRR) affects crop productivity and resilience to climate-driven factors. The availability of good-quality seeds of high-yielding varieties with superior genetic purity is essential for high production under different agro-climatic conditions in any crop. Farm productivity has a significant positive connection with farmer’s prosperity and livelihood, and the timely availability of high-quality seeds of high-yielding varieties plays a vital role in it (Singh, 2015; Chand et al., 2022b). Timely availability of quality seed alone can increase the yield by 15%–20%, and it may go up to 45% with proper management practices (https://seednet.gov.in/). Several challenges have been reported in the past, mainly related to quality seed production, which adversely affected the expansion of cultivated areas under the particular crop (Chauhan et al., 2017; 2021).

In the present study, we have analyzed the BS demand and production trend during the last 24 years (1998–1999 to 2021–2022) in India. We explained the factors affecting BS demand and production and the possible reasons for the shortfall in BS production. The VRR is also calculated in the lucerne for the first time. In addition, we have predicted the potential production of foundation and certified seed from the produced BS. The present study highlights the existing challenges in BS production. It also points towards the need to reframe our breeding programs to develop new high-yielding varieties.

## 2 Materials and methods

The BS indent and production data of different lucerne varieties under the seed chain in India were collected from the AICRP FC&U (Anonymous, 1999; 2000; 2001; 2002; 2003; 2004; 2005; 2006; 2007; 2008; 2009; 2010; 2011; 2012; 2013; 2014; 2015; 2016; 2017; 2018; 2019; 2020; 2021; 2022) located in ICAR–Indian Grassland and Fodder Research Institute, Jhansi (India). The raw data were compiled, analyzed, and interpreted to express vividly the status of BS demand and production of varieties in the seed chain since 1998–1999. Microsoft Excel (2013 version) was used for preparing different figures, such as BS demand and production trends, and the contribution of major institutions to BS allocation and production. In addition, VRR for the last 3 years (2019–2020 to 2021–2022) was also calculated using the following formula: VRR= (A/B) × 100; where A = indent of given varieties (kg) for the calculated years; B = total indent of all varieties of the given crop (kg) for the calculated years. It expressed the contribution of recently released varieties (varietal age <5 years) in BS demand. Likewise, foundation seed, certified seed, and area under certified seed of lucerne varieties at farmer’s fields were also predicted based on available 2021–2022 BS production.

## 3 Results

### 3.1 Aggregate breeder seed demand and production status

**Figure 1:**
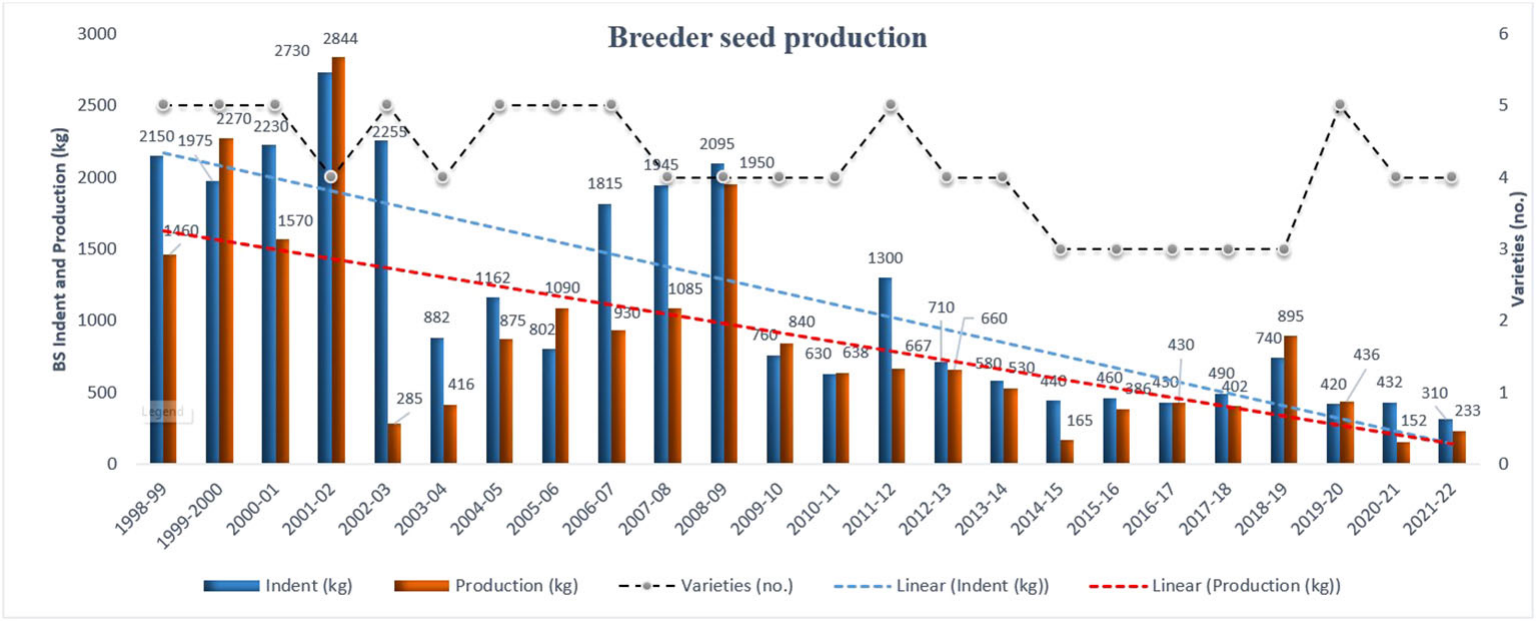
Lucerne breeder seed indent, production, and number of varieties over the years in seed chain during the last 24 years in India. Dotted blue and red lines are linear regression and express the declining trend of both BS indent and production, respectively.

In lucerne, during the last 24 years, the BS demand gradually decreased with few fluctuations in trend, and only three to five notified varieties were in the seed chain for any particular year (**Figure 1**). For ease of calculation and interpretation, the 24 years were divided into six blocks of 4 years each. For instance, from 1998–1999 to 2001–2002, DAC indented 9,085 kg of lucerne varieties for BS production to AICRP FC&U and was considered base year block (**Supplementary Table S1**). During 2002–2003 to 2005–2006, the BS indent was 5,101 kg, a reduction of 43.85% over the base year block. Likewise, the BS indent was reduced by 27.19%, 64.56%, 79.97%, and 79.06% over the base year block from 2006–2007 to 2009–2010, 2010–2011 to 2013–2014, 2014–2015 to 2017–2018, and 2018–2019 to 2021–2022, respectively.

In 1998–1999, BS production was 1,460 kg against the indent 2,150 kg, and the deficit was 690 kg (32.09%). However, BS indent was reduced by 85.58% in 2021–2022 compared to 1998–1999 (**Figure 1**). The BS production was 233 kg for the indent 310 kg and was a deficit of 77 kg (24.84%) in 2021–2022. The BS production of lucerne varieties could not match their respective allocations during the last 24 years except for a few years like 1999–2000 (+295 kg), 2001–2002 (+144 kg), 2005–2006 (+288 kg), 2009–2010 (+80 kg), 2010–2011 (+8 kg), 2016–2017 (equal), 2018–2019 (+155 kg), and 2019–2020 (+16 kg) (**Figure 1**). It is worth mentioning that institutions take up the BS production only after getting the demand; hence, BS production directly connects with the BS indent in any particular year. Less the BS indent would be BS production. BS production was reduced by 67.26%, 41.00%, 69.36%, 83.02%, and 78.93% during 2002–2003 to 2005–2006, 2006–2007 to 2009–2010, 2010–2011 to 2013–2014, 2014–2015 to 2017–2018 and 2018–2019 to 2021–2022, respectively, in lucerne over the base year block (**Supplementary Table S1**).

### 3.2 Varietal diversification, their BS indent, and production status

In India, only 14 varieties have been released and notified in lucerne since 1978 under the National Agricultural Research System (NARS); however, only nine varieties have been incorporated in the seed chain since 1998–1999 (Roy et al., 2020). Detailed information, *viz.*, breeding method, mother institute, and adoption area, related to indented varieties is presented in **Table 1**. However, only three to five lucerne varieties were in the seed chain in any particular calendar year during the last 24 years (**Figure 1**), and varietal BS demand and production over the years are presented in **Supplementary Table S2**. Varietal diversification is essential for increasing crop production, suitability of a variety in a specific cropping system, more buffering capacity to biotic and abiotic stresses, and more choice for the farmers based on their available resources.

**Table 1:** Detailed description of the lucerne varieties indented during the last 24 years in India (Data source: Roy et al., 2020).

S.N.	Variety*	Year of notification	Breeding method/source	Parent institute	GFY (000 kg/ha)	Area of adoption	Specific features
1.	Type–9	1978	Mass selection from the lucerne germplasm	CCSHAU, Hisar	45–50	Haryana, Rajasthan, Gujarat, Himachal Pradesh and Delhi under irrigated conditions	Perennial
2.	CO-1	1982	Mass selection from local material of Coimbatore	TNAU, Coimbatore	100–120	Tamil Nadu	Perennial, free from Cuscuta
3.	LLC-5	1984	Selected clones from Kutchh area of Gujarat after three cycles of recurrent selection	PAU, Ludhiana	70–75	Gujarat, Maharashtra, Andhra Pradesh, Rajasthan, Himachal Pradesh, Punjab and Haryana	Annual, moderately resistant to downy mildew
4.	Anand-2	1984	Pure line selection from the material collected from Kutchh areas of Gujrat	GAU, Banaskantha	70–75	Gujarat, Rajasthan and Maharashtra	Annual type; vigorous growth, suitable for seasonal and annual cultivation
5.	Anand-3	1995	Introduction from GAU, Anand (Gujarat)	CSKHPKV, Palampur	45–50	Cold dry zone of Kinnaur and Lahul and Spiti valley of Himachal Pradesh	Perennial, resistant to logging and frost and highly responsive to Phosphate fertilizer
6.	RL-88	1996	Selection from Ahmednagar local Lucerne	MPKV, Rahuri	100–120	Lucerne growing irrigated areas in the country	Perennial, quick re-growth, more vigorous than any other varieties
7.	AL-3	2009	Pure line selection from the material collected from Kutchh areas of Gujrat	AAU, Anand	100–120	Sub-tropical areas of Gujrat and Maharashtra	Perennial, oblong dark green leaves; free from major diseases
8.	RBB 07-01	2016	Composite of seven cultivars	SKRAU, Bikaner	160–180	North West zone of India	Perennial, high crude protein
9.	TNLC-14	2019	Polycross derivative involving CO 1	TNAU, Coimbatore	45–50	Telangana, Andhra Pradesh, Tamil Nadu, Karnataka	Perennial

*Type-9 is also known as T-9, LLC-5 as LL composite-5, RBB 07-01 as Krishna; Anand-2 as GAUL-1, RL-88 as RLS-88, CO-3 as TNLC-14; GFY: potential green fodder yield (000 kg/ha).

Five lucerne varieties were in the seed chain during 1998–1999 to 2001–2002, and BS indent was highest for Anand-2, followed by Type-9 and RL-88 contributing 42.71%, 35.17%, and 10.40%, respectively (**Table 2**). Likewise, BS indent was maximum for Anand-2 followed by Type-9 and contributed 40.07% and 23.88%, respectively, during 2002–2003 to 2005–2006. Variety Anand-2 had maximum BS indent and shared more than 50% contribution to the total BS indent after each 4-year interval since 2006–2007. Overall, Anand-2 (53.11%) had contributed the highest share in BS indent, followed by Type-9 (19.44%) and RL-88 (13.60%) during the last 24 years.

**Table 2:** Varietal breeder seed indent and production status of lucerne varieties and their contribution after every 4 years during the last 24 years in India.

Years	Status*	Type-9	CO-1	LLC-5	Anand-2	Anand-3	RL-88	AL-3	RBB 07-01	CO-3
1998–1999 to 2001–2002	Indent	3,195 (35.17)	770 (8.48)	295 (3.25)	3,880 (42.71)		945 (10.40)			
	Production	1,440 (17.68)	460 (5.65)	90 (1.11)	5,349 (65.68)		805 (9.88)			
	+/−	−1,755	−310	−205	+1,469		−140			
2002–2003 to 2005–2006	Indent	1,218 (23.88)	646 (12.66)	123 (2.41)	2,044 (40.07)		1070 (20.98)			
	Production	516 (19.35)	285 (10.69)	65 (2.44)	1,325 (49.70)		475 (17.82)			
	+/−	−702	−361	−58	−719		−595			
2006–2007 to 2009–10	Indent	780 (11.79)	115 (1.74)		4,355 (65.84)	70 (1.06)	1,195 (18.07)	100 (1.51)		
	Production	90 (1.87)	110 (2.29)	20 (0.42)	3,895 (81.06)		590 (12.28)	100 (2.08)		
	+/−	−690	−5	20	−460	−70	−605			
2010–2011 to 2013–14	Indent	200 (6.21)	300 (9.32)		1,750 (54.35)	50 (1.55)	410 (12.73)	510 (15.84)		
	Production	118 (4.73)	100 (4.01)		1750 (70.14)		310 (12.42)	217 (8.70)		
	+/−	−82	−200		−	−50	−100	−293		
2014–2015 to 2017–2018	Indent				1,400 (76.92)	280 (15.38)	70 (3.85)	70 (3.85)		
	Production				1,190 (86.04)	105 (7.59)	73 (5.28)	15 (1.08)		
	+/−				−210	−175	+3	−55		
2018–2019 to 2021–2222	Indent				1,305 (68.61)	10 (0.53)	82 (4.31)	230 (12.09)	185 (9.73)	90 (4.73)
	Production				1,055 (61.48)	10 (0.58)	283 (16.49)	230 (13.40)	73 (4.25)	65 (3.79)
	+/−				−250	−	+201	−	−112	−25
Total	Indent	5,393 (19.44)	1831 (6.60)	418 (1.51)	14,734 (53.11)	410 (1.48)	3,772 (13.60)	910 (3.28)	185 (0.67)	90 (0.32)
	Production	2,164 (10.20)	955 (4.50)	175 (0.83)	14,564 (68.67)	115 (0.54)	2,536 (11.96)	562 (2.65)	73 (0.34)	65 (0.31)
	+/−	−3,229	−876	−243	−170	−295	−1,236	−348	−112	−25
	% change	−59.87	−47.84	−58.13	−1.15	−71.95	−32.77	−38.24	−60.54	−27.78

^*^+/- indicates surplus or deficit BS production (kg) compared to allocation; values in parentheses represents the percent contribution of each variety to the total BS indent and production.

As far as varietal BS production is concerned, Anand-2 contributed maximum, followed by Type-9 from 1998–1999 to 2005–2006 (**Table 2**). Likewise, Anand-2 contributed maximum, followed by RL-88 to the total BS production during 2006–2007 to 2009–2010, 2010–2011 to 2013–2014, and 2018–2019 to 2021–2022, respectively. Anand-3 (7.59%) contributed second highest after Anand-2 (86.04%) during 2014–2015 to 2017–2018. Overall, Anand-2 had the highest share with the value of 68.67%, followed by RL-88 (11.96%) and Type-9 (10.20%) in the total BS production during the last 24 years.

### 3.3 Disparity in varietal BS indent and production

Allocated production centers failed to meet the BS production targets against their indent in lucerne since 1998–1999 (**Table 3**). For instance, allocated centers produced 8,144 kg BS against the indent 9,085 kg with a net deficit of 941 kg (10.36%) during 1998–1999 to 2001–2002 (**Supplementary Table S1**). Likewise, BS production was net deficit of 2,435 kg (47.74%) as against the total BS indent, i.e., 5,101 kg from 2002–2003 to 2005–2006. However, after that, the gap between BS production and BS indent was narrowed down by extensive efforts of the ICAR, AICRP FC&U, concerned breeders, and parent institutes. For example, BS production was a net deficit of 27.36%, 22.52%, 24.01%, and (9.78%) against the allocated quantity from 2006–2007 to 2009–2010, 2010–2011 to 2013–2014, 2014–2015 to 2017–2018 and 2018–2019 to 2021–2022, respectively. The varietal BS production scenario indicates that only Anand-2 was produced in surplus (+1,469 kg) against the indent (3,880 kg), whereas other indented varieties could not meet the BS indent during 1998–1999 to 2001–2002 (**Table 2**). For instance, Type-9 had a deficit of 1,755 kg against the BS indent of 3,195 kg. From 2002–2003 to 2005–2006, BS production was less than its demand in all indented lucerne varieties; for instance, there was a net deficit of 719 kg and 702 kg against the indent of 2,044 kg and 1,218 kg in Anand-2 and Type-9, respectively. Except for LLC-5, BS demand for other indented varieties could not be met from 2006–2007 to 2009–2010. For example, Type-9 and RL-88 had a net deficit of 690 kg and 605 kg against their BS indent, respectively. Similarly, BS production was less than BS indent for all indented varieties except Anand-2, where production was equal to indent (1,750 kg) during 2010–2011 to 2013–2014. From 2014–2015 to 2017–2018, allocated centers could not meet the BS demand for indented varieties except RL-88, which was in surplus (+3.0 kg) against the allocation. BS demand was fulfilled for Anand-3 and AL-3, whereas RL-88 was produced in surplus (+201 kg) during 2018–2019 to 2021–2022. Overall, the BS demand for lucerne varieties could not be fulfilled during the last 24 years. However, the total BS production of the most popular variety, i.e., Anand-2, was 14,564 kg against the indent (14,734 kg), and only 170 kg was a deficit.

**Table 3:** The breeder seed allocation and production status of indented lucerne varieties to the different production centres and their net BS balance during the last 24 years in India (data source: Anonymous, 1999; 2000; 2001; 2002; 2003; 2004; 2005; 2006; 2007; 2008; 2009; 2010; 2011; 2012; 2013; 2014; 2015; 2016; 2017; 2018; 2019; 2020; 2021; 2022).

Variety	Allocated center^*^	Allocation (kg)	Production (kg)	Net deficit (kg)	Deficit (%)
Anand-2	AAU, Anand	14,734	14,564	−170	−1.15
CO-1	TNAU, Coimbatore	1,831	955	−876	−47.84
LLC-5	PAU, Ludhiana	418	175	−243	−58.13
RL-88	MPKV, Rahuri	3,772	2,536	−1,236	−32.77
Type-9	CCSHAU, Hisar	380	208	−172	−45.26
	IGFRI, Jhansi	30	0	−30	−100.00
	MPKV Rahuri	400	0	−400	−100.00
	UAS Bangalore	170	0	−1.70	−100.00
	AAU, Anand	1,218	516	−702	−57.64
	Gandhinagar, RSFPD	2,175	1,040	−1,135	−52.18
	NDDB, Anand	1,002	400	−602	−60.08
Anand-3	AAU, Anand	400	175	−225	−56.25
	CSKHPKV, Palampur	70	0	−70	−100.00
AL-3	AAU, Anand	850	502	−348	−40.94
RBB-07-01	SKRAU, Bikaner	185	73	−112	−60.54
CO-3	TNAU, Coimbatore	90	65	−25	−27.78
	Total	27,743	21,209	−6,534	−23.55

*AAU, Anand Agricultural University, Anand; TNAU, Tamil Nadu Agricultural University, Coimbatore; PAU, Punjab Agricultural University, Ludhiana, MPKV, Mahatma Phule Krishi Vidyapeeth, Rahuri; Chaudhary Charan Singh Haryana Agricultural University, Hisar; IGFRI, Indian Grassland and Fodder Research Institute, Jhansi; UAS, University of Agricultural Sciences, Bangalore; RSFPD, Regional Station for Forage Production and Demonstration, Gandhinagar; NDDB, National Dairy Development Board, Anand; CSKHPKV, Chaudhary Sarwan Kumar Himachal Pradesh Krishi Vishvavidyalaya, Palampur, SKRAU, Swami Keshwanand Rajasthan Agricultural university, Bikaner.

### 3.4 BS producing centers, indent, and production status

Only three SAUs, Anand Agriculture University (AAU)–Anand, Mahatma Phule Krishi Vidyapeeth (MPKV)–Rahuri, and Tamil Nadu Agriculture University (TNAU)–Coimbatore, have consistently participated and also shared 91.06% to the total BS indent in lucerne from 1998–1999 to 2021–2022 (**Supplementary Figure S1**). The AAU–Anand has maximum contribution (74.29%) to the BS indent, followed by MPKV–Rahuri (11.96%) and TNAU–Coimbatore (4.81%) from 1998–1999 to 2021–2022. Overall, allocating centers, *viz.*, AAU–Anand, MPKV–Rahuri, TNAU–Coimbatore, RSFPD–Gandhinagar, and NDDB–Anand could not meet the BS demand in lucerne from 1998–1999 to 2021–2022.

The AAU–Anand could not meet the BS demand of lucerne varieties; for example, Anand-2 had a deficit of 170 kg, Type-9 of 702 kg, Anand-3 of 225 kg, and AL-3 of 348 kg against the BS indent during the last 24 years (**Table 3**). Similarly, TNAU–Coimbatore’s production figures also indicated less CO-1 (876 kg) and CO-3 (25 kg) against their allocation. There was a deficit production of 243 kg against the indent (418 kg) for variety LLC-5 by PAU–Ludhiana and a deficit of 1,236 kg against the indent (3772 kg) for variety RL-88 by MPKV–Rahuri. Similarly, other centers could not produce BS in sufficient amounts to meet the BS demand of the allocated varieties (**Table 3**).

### 3.5 Varietal replacement rate

**Figure 2:**
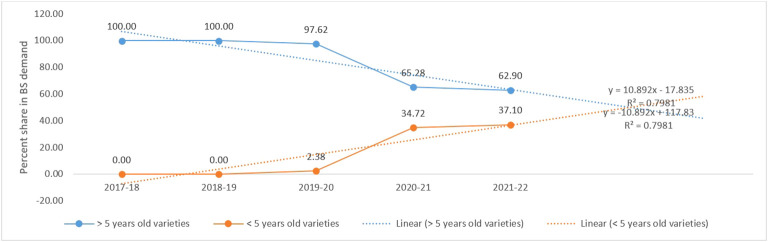
Percent contribution of lucerne varieties (<5 years and >5 years old) to the total BS indent during the last 5 years (2017–2018 to 2021–2022).

Since 2017–2018, the percent share of old varieties (>5 years) has declined substantially and *vice versa* for <5-year-old varieties (**Figure 2**). Two varieties, having less than 5 years of varietal age, were indented (275 kg) and shared 23.67% of the total indent (**Table 4**). Likewise, the varieties having <15 years of age (only three varieties) shared 38.30%; however, the varieties having more than 15 years of age (only three varieties) still had a 61.70% contribution to the total BS indent.

**Table 4:** Varietal replacement rate (VRR) in lucerne during last 3 years (2019–2020 to 2021–2022) in India under public sector.

Number of total notified varieties	No. of varieties in seed chain	Total BS indent (kg)	Varieties < 5 years old			Varieties < 15 years old			Varieties > 15 years old		
			No.	Indent (kg)	% share in total indent	No.	Indent (kg)	% share in total indent	No.	Indent (kg)	% share in total indent
14	6	1,162	2	275	23.67	3	445	38.30	3	717	61.70

### 3.6 Prediction of foundation and certified seeds

Foundation seed is the progeny of BS, whereas certified seed is derived from foundation seed. Certified seed, also known as commercial seed, is available to farmers with 99% genetic purity. The quantity of the foundation seed and certified seed produced was calculated based on conversion of total breeder seeds at 1:26 seed multiplication ratio (Chauhan et al., 2017). In this study, the total BS production in lucerne crop was 233 kg in four different varieties, *viz.*, Anand-2 (175 kg), RBB-07-01 (23 kg), AL-3 (20 kg), and CO-3 (15 kg) in *Rabi* 2021–2022 (**Supplementary Table S2**). The foundation seed production would be 6058 kg in 2022–2023 if the seed chain functions 100% effectively and all the practices are followed correctly (**Table 5**). Likewise, certified seed production would be 157,508 kg in the subsequent year (2023–2024) and will cover 6.3 thousand hectares of area in the farmer’s field under fodder lucerne.

**Table 5:** Breeder, foundation, and certified seed demand and prediction of foundation and certified seed in lucerne from the available breeder seed in India.

Crop	Seed rate (kg/ha) for		SMR^*^	Approx. area^*^ (Mha)	Seed demand (kg)			Seed production (kg)			Estimated area covered (000 ha) (2024–2025)
	Fodder production	Seed production			BS (2021–2022)	FS (2022–2023)	CS (2023–2024)	BS (2021–2022)	FS (2022–2023)	CS (2023–2024)	
Lucerne	25	15	26	1.0	36,500	950,000	24,750,000	233	6,058	157,508	6.30

*According to Chauhan et al., 2017.

SMR, seed multiplication ratio; BS, breeder seed; FS, foundation seed; CS, certified seed.

## 4 Discussion

Breeder seed production is vital for maintaining the long-term seed chain and is the prime responsibility of public sector institutions and agencies (Vishnu et al., 2013; Prasad et al., 2022). The Indian seed program recognizes three generation system: breeder, foundation, and certified seeds. It assures adequate quality standard in the seed multiplication chain and also maintains the genetic purity of a variety as it moves from the breeder to the last stakeholder, i.e., farmers (Mishra et al., 2022; Yadav et al., 2022). Certified seeds are commercialized and sold in the market to farmers/agencies for raising the crop and its utilization.

In lucerne, the BS demand for indented varieties has declined substantially since 1998–1999. The present study observed that BS indent has reduced by 85.58% from 1998–1999 to 2021–2022. However, increasing trends of BS demand have been reported in food crops like wheat (Krishna et al., 2016), rice (Prasad et al., 2022), barley (Vishnu et al., 2013), and pulses (Parihar and Dixit, 2016). There might be few probable but imperative reasons that could explain the declining lucerne BS demand in the country such as (1) shifting of cultivated lucerne area into different fodder crops, (2) disparity in BS production and demand, (3) unrealistic and extremely high BS demand by the indenters, (4) extensive efforts of private seed companies in seed production and direct selling to the farmers, and (5) presence of the unorganized seed sector.

**Figure 3:**
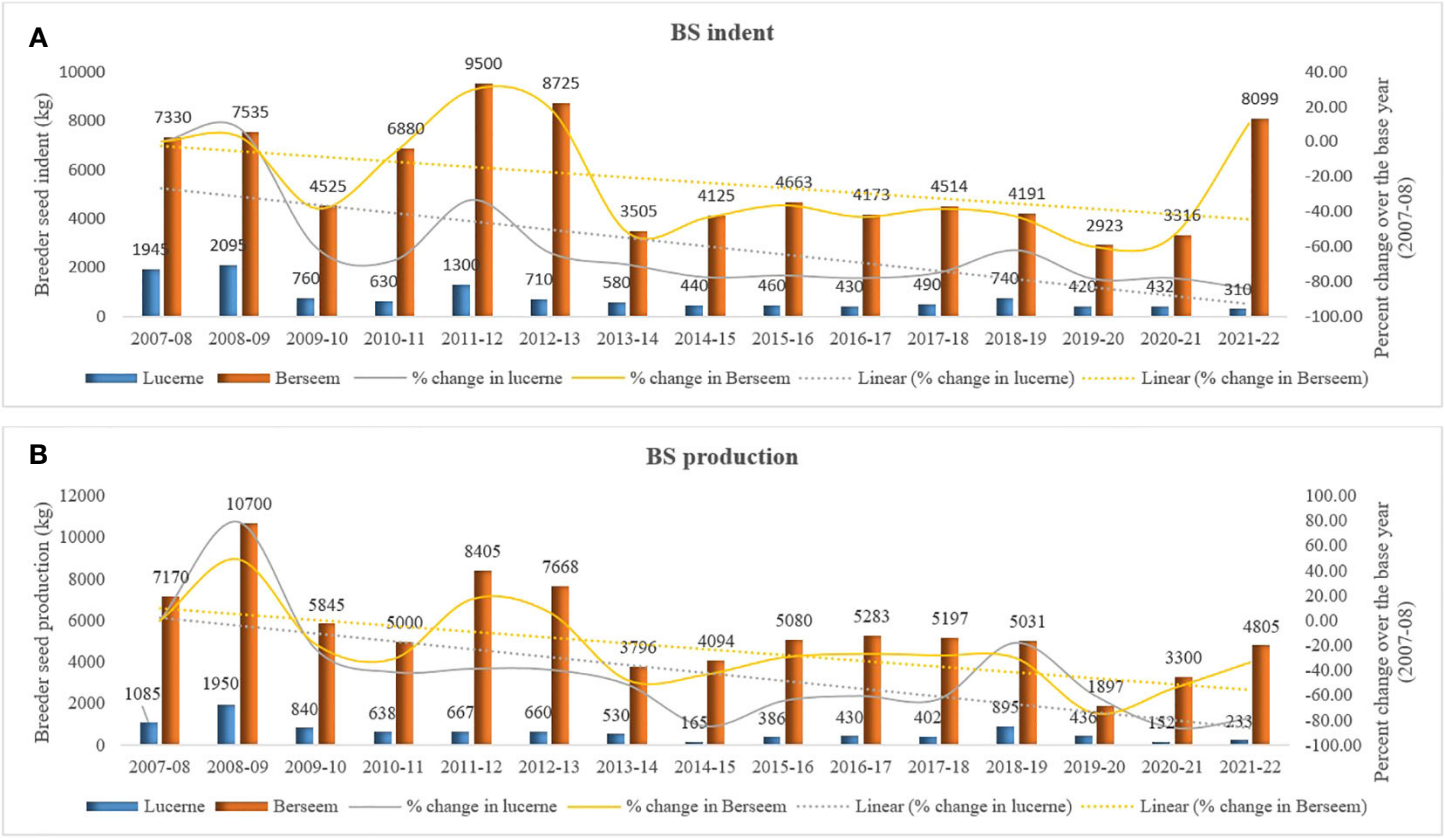
Comparison between BS demand and production of lucerne **(A)** and berseem **(B)** crops during the last 15 years (2007–2008 to 2021–2022).

First, lucerne cultivated area has been occupied by other fodder crops, more specifically berseem, ryegrass, and multicut sorghum over time; however, berseem BS demand during the last 15 years indicated that there is not much area expansion in the berseem (**Figure 3**). Both lucerne and berseem crops are important leguminous fodder crops of *Rabi* season in India; however, lucerne is superior over berseem in nutritional quality and dry matter production (Nasrullah et al., 2015; Kaithwas et al., 2020; Roy et al., 2020). In addition, the country’s berseem BS demand and production is almost constant from 2013–2014 to 2020–2021. Both the crops have different cultivable agro-ecological areas; for instance, berseem is mainly grown in the states of Punjab, Haryana, Uttar Pradesh, and parts of Rajasthan and Madhya Pradesh; however, lucerne cultivation areas are Gujrat, Rajasthan, Maharashtra, Karnataka, and Tamil Nadu. Lucerne crop is mostly preferred in those areas where the water supply is inadequate and winter period is short (https://aicrponforagecrops.icar.gov.in/). Furthermore, it has been observed that a few areas of lucerne (Rajasthan, Uttar Pradesh and *Malwa* region of Madhya Pradesh) have been converted into berseem cultivation due to the availability of seed with affordable lower prices. In addition, ryegrass and multicut sorghum are also encroaching on the traditional acreages of lucerne in Gujarat and Madhya Pradesh (personal observation).

Second, production centers could not produce sufficient BS to meet the demand; therefore, indenters indented less BS demand or changed the fodder crop for the same use in the subsequent years since 1998–1999. However, the production center almost fulfilled the BS demand of the most indented variety, i.e., Anand-2, in the last 24 years (**Table 2**). In addition, when a production center could not meet the BS demand of a particular variety, the indent gradually shifted to another notified variety. For example, BS production of Type-9 and Anand-3 was too low to meet the BS demand; therefore, the indent was shifted to Anand-2 and RL-88 in the subsequent years (**Supplementary Table S2**). Anand-2, being an annual cultivar, is mostly preferred in the northern parts of India such as western Punjab and Haryana where it provides high green acreage in five to six cuts spread over October to June. On the other side, RL-88—being a perennial variety—is regularly preferred by farmers in central and southern states of India like Maharashtra, Gujrat, and Tamil Nadu and could be maintained for up to 3 years in farmer’s field, providing high production of green forage.

Third, indenters placed unrealistic and unwarranted BS demand, and production centers produced substantial amounts of BS against the indent; however, indenters could not uplift the BS from the centers due to various reasons for many years (personal observation). This matter was discussed at various platforms of ICAR and DAC, and thereafter, indenters started to place realistic BS demand to the DAC. Therefore, BS indent declined substantially after 2008–2009, and proper seed chain was followed for foundation and certified seed production.

Fourth, private seed companies’ contribution to lucerne seed production has increased substantially over the last 15 years. More than 500 private sector companies actively participate in seed production in various crops and have a share of >80% in India’s seed sale and are involved mostly in low-volume and high-value crops (Agrawal, 2012; HanChinal, 2012; Chauhan et al., 2016). However, only 10–12 private seed companies, *viz.*, Alamdar Seeds, Foragen Seeds, and Kisan Kutch Seeds, are actively involved in lucerne seed production and marketing of un-certified seeds to the farmers. Their robust extension programs, promotion strategies, and direct participation of farmers provide an edge over government programs to disseminate their technologies to the farmer’s field. Private companies contract with farmers to produce seed at a large scale, where farmers get quality seed and other inputs from seed companies (Chauhan et al., 2016). These companies purchase the farm produce at reasonable rates and sell it to the markets after processing or grading at competitive rates. Fifth, the informal seed sector, particularly farmers who produce seed at their farms, distribute it among their relatives and sell it to other growers in nearby villages (Hiremath et al., 2020).

Crop production is adversely affected by climatic conditions such as abiotic (rainfall pattern and intensity, low and high temperature, drought, salinity, alkalinity, etc.) and biotic (disease and pest infestation) factors (Joshi et al., 2007; Roy et al., 2016; Chand et al., 2022a). In India, lucerne BS productivity is low (180–250 kg/ha), and several factors such as unprecedented and erratic rainfall patterns, high isolation distance, severe inbreeding depression on selfing, increased dependency on bee visits for tripping, and high sensitivity to abiotic and biotic stresses.

The development and deployment of high-yielding and stable varieties are the need of the hour to increase the production and productivity of any crop, and VRR is an indicator of the dissemination of genetic progress. (Singh et al., 2020; Chauhan et al., 2021; Prasad et al., 2022). In the present study, the contribution of recently released and notified lucerne varieties (<5 years old) is moderate, and the contribution of old varieties (>15 years old) is very high in the last 3 years (2019–2020 to 2021–2022). Wheat has the highest VRR among the crops, followed by mung bean and chickpea in India. In wheat, varieties notified during the last 5 and 10 years shared 45.3% and 74.0%, respectively, during 3 years (2017–2018 to 2019–2020) (Singh et al., 2020). In addition, the percentage share of varieties over 5 years of age has been declining gradually since 2017–2018, and the contribution might be more than 50% in the coming years at this pace. India requires 36,500 kg BS per annum, if 100% seed replacement rate is followed, to cover the existing 1 Mha area (Chauhan et al., 2017). However, available BS can meet only 0.6% requirement of the commercial seed, and 0.4% would be met by informal seed supply chain including private seed companies, farm saved seeds, and non-certified seeds from local markets. Therefore, there is an urgent need to develop breeding programs to improve genetic gain using conventional and non-conventional approaches and develop more genotypes with high green forage yield and nutritional superiority. Conventional approaches mean classical breeding tools like introduction, selection, hybridization, and pedigree. Non-conventional approaches include biotechnological tools such as marker-assisted selection (MAS), genetic engineering, tissue culture, embryo rescue, and genetic transformation.

## 5 Conclusion

In India, low productivity of forage crops is a major concern in which timely availability of sufficient quality seed is a foremost factor. The BS is a vital component of the seed chain and decides the time-bound availability of certified seed to the farmers. The BS demand for improved varieties for different agro-climatic conditions and cropping systems needs to be improved in lucerne. The BS demand will increase with the certified seed demand. The government would act to promote lucerne growing and the use of certified (public) seed. Genetic progress is another, but important, subject. In addition, it must be ensured that the production center should produce an adequate amount of BS to meet the demand.

## Data availability statement

Publicly available datasets were analyzed in this study. This data can be found here: https://aicrponforagecrops.icar.gov.in.

## Author contributions

SC: Conceptualization, Data curation, Formal analysis, Investigation, Methodology, Project administration, Resources, Software, Supervision, Validation, Visualization, Writing – original draft, Writing – review & editing. AKR: Conceptualization, Data curation, Formal analysis, Resources, Validation, Writing – review & editing, Investigation, Project administration, Supervision, Writing – original draft. RKA: Conceptualization, Formal analysis, Resources, Supervision, Writing – review & editing. TS: Conceptualization, Data curation, Validation, Visualization, Writing – review & editing. DKY: Data curation, Investigation, Project administration, Supervision, Validation, Visualization, Writing – review & editing. VKY: Conceptualization, Data curation, Writing – review & editing. AC: Conceptualization, Formal analysis, Validation, Writing – review & editing. SK: Software, Writing – review & editing. DRM: Supervision, Formal analysis, Writing – review & editing.

## Supplementary material

Supplementary Figure 1 Histogram highlighting the total BS production against the indent at major centers and their percent contribution to the lucerne BS production centers during the last 24 years in India.
