# Supplementary figures and images for: Twenty-four years lucerne (Medicago sativa L.) breeder seed production in India: a retrospective study
Source: Front Plant Sci. 2023 Oct 26;14:1259967. doi: 10.3389/fpls.2023.1259967 (PMC10640986; doi:10.3389/fpls.2023.1259967)

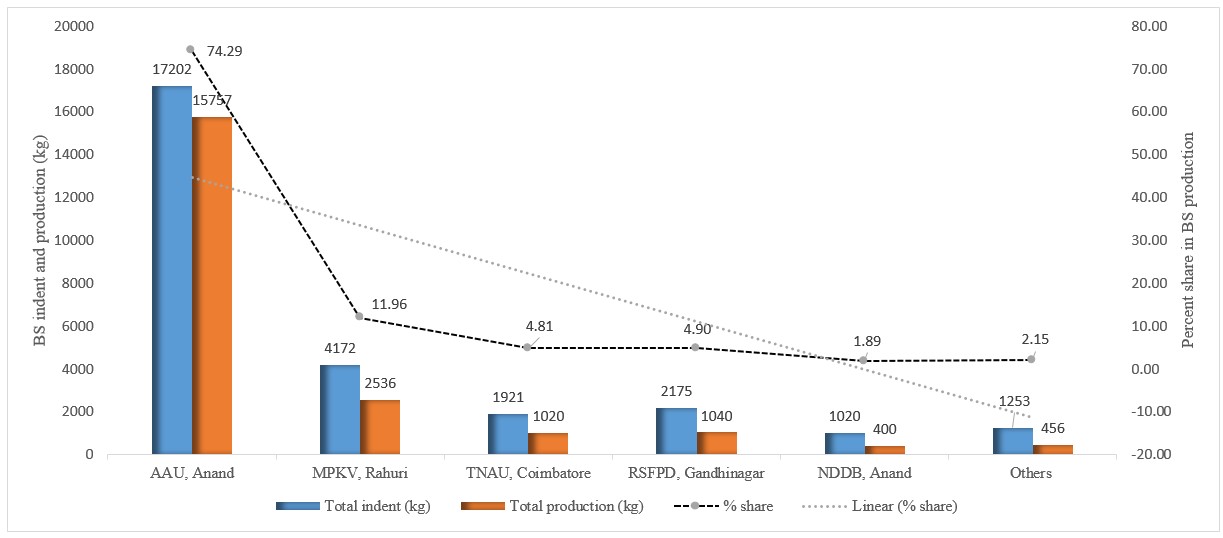

Supplement: Supplementary Figure 1 — Histogram highlighting the total BS production against the indent at major centers and their percent contribution to the lucerne BS production centers during the last 24 years in India. [file Image_1.jpg]
