# Supplementary material for: Twenty-four years lucerne (Medicago sativa L.) breeder seed production in India: a retrospective study
Source: Front Plant Sci. 2023 Oct 26;14:1259967. doi: 10.3389/fpls.2023.1259967 (PMC10640986; doi:10.3389/fpls.2023.1259967)
Supplement: Supplementary file 2 [file Table_1.docx]

**Supplementary Table S1.** Percent change of lucerne breeder seed indent and production at four years intervals in India during the last 24 years.

| **Years^#^** | **Indent** | | **Production** | | **Overall** | |
| --- | --- | --- | --- | --- | --- | --- |
|  | **Quantity (kg)** | **% change** | **Quantity**  **(kg)** | **% change** | **Surplus/Deficit (kg)** | **Surplus/Deficit (%)** |
| 1998–99 to 2001–02 | 9085 | - | 8144 | - | -941 | -10.36 |
| 2002–03 to 2005–06 | 5101 | -43.85 | 2666 | -67.26 | -2435 | -47.74 |
| 2006–07 to 2009–10 | 6615 | -27.19 | 4805 | -41.00 | -1810 | -27.36 |
| 2010–11 to 2013–14 | 3220 | -64.56 | 2495 | -69.36 | -725 | -22.52 |
| 2014–15 to 2017–18 | 1820 | -79.97 | 1383 | -83.02 | -437 | -24.01 |
| 2018–19 to 2021–22 | 1902 | -79.06 | 1716 | -78.93 | -186 | -9.78 |
| Total | 27743 | - | 21209 | - | -6534 | -23.55 |

^#^first four years block (1998–99 to 2001–02) was considered as base year block and percent change was calculated over the base year block
