# Supplementary material for: Twenty-four years lucerne (Medicago sativa L.) breeder seed production in India: a retrospective study
Source: Front Plant Sci. 2023 Oct 26;14:1259967. doi: 10.3389/fpls.2023.1259967 (PMC10640986; doi:10.3389/fpls.2023.1259967)
Supplement: Supplementary file 3 [file Table_2.docx]

**Supplementary Table S2.** Breeder seed production status of indented lucerne varieties during the last 24 years.

| Year | DAC Indent and Production (kg) | **Lucerne varieties under seed chain** | | | | | | | | |
| --- | --- | --- | --- | --- | --- | --- | --- | --- | --- | --- |
|  |  | Type-9 | CO-1 | LLC-5 | Anand-2 | Anand-3 | RL-88 | AL-3 | RBB 07-01 | CO-3 |
|  |  | **Notification year** | | | | | | | | |
|  |  | 1978 | 1982 | 1984 | 1984 | 1995 | 1996 | 2009 | 2016 | 2019 |
| 1998-99 | Indent | 1110 | 100 | 20 | 880 |  | 40 |  |  |  |
|  | Production | 160 | 1 00 | 20 | 1140 |  | 40 |  |  |  |
| 1999-20 | Indent | 1020 | 20 | 20 | 815 |  | 100 |  |  |  |
|  | Production | 900 | 100 | 20 | 1150 |  | 100 |  |  |  |
| 2000-01 | Indent | 1065 | 275 | 20 | 530 |  | 340 |  |  |  |
|  | Production | 380 | 80 | 20 | 890 |  | 200 |  |  |  |
| 2001-02 | Indent |  | 375 | 235 | 1655 |  | 465 |  |  |  |
|  | Production |  | 180 | 30 | 2169 |  | 465 |  |  |  |
| 2002-03 | Indent | 780 | 390 | 115 | 520 |  | 450 |  |  |  |
|  | Production | 255 |  | 30 |  |  |  |  |  |  |
| 2003-04 | Indent | 125 | 102 |  | 435 |  | 220 |  |  |  |
|  | Production | 161 | 110 |  |  |  | 145 |  |  |  |
| 2004-05 | Indent | 188 | 102 | 8 | 594 |  | 270 |  |  |  |
|  | Production | 100 | 100 | 15 | 600 |  | 60 |  |  |  |
| 2005-06 | Indent | 125 | 52 |  | 495 |  | 130 |  |  |  |
|  | Production |  | 75 | 20 | 725 |  | 270 |  |  |  |
| 2006-07 | Indent | 510 | 65 |  | 830 |  | 410 |  |  |  |
|  | Production |  | 60 | 20 | 850 |  |  |  |  |  |
| 2007-08 | Indent | 60 | 50 |  | 1425 |  | 410 |  |  |  |
|  | Production |  | 50 |  | 945 |  | 90 |  |  |  |
| 2008-09 | Indent | 50 |  |  | 1700 | 70 | 275 |  |  |  |
|  | Production | 50 |  |  | 1700 |  | 200 |  |  |  |
| 2009-10 | Indent | 160 |  |  | 400 |  | 100 | 100 |  |  |
|  | Production | 40 |  |  | 400 |  | 300 | 100 |  |  |
| 2010-11 | Indent | 50 |  |  | 450 |  | 110 | 20 |  |  |
|  | Production | 18 |  |  | 450 |  | 150 | 20 |  |  |
| 2011-12 | Indent | 50 | 300 |  | 450 |  | 200 | 300 |  |  |
|  | Production |  | 100 |  | 450 |  | 60 | 57 |  |  |
| 2012-13 | Indent | 100 |  |  | 450 |  | 50 | 110 |  |  |
|  | Production | 100 |  |  | 450 |  |  | 110 |  |  |
| 2013-14 | Indent |  |  |  | 400 | 50 | 50 | 80 |  |  |
|  | Production |  |  |  | 400 |  | 100 | 30 |  |  |
| 2014-15 | Indent |  |  |  | 360 |  | 10 | 70 |  |  |
|  | Production |  |  |  | 150 |  |  | 15 |  |  |
| 2015-16 | Indent |  |  |  | 360 | 90 | 10 |  |  |  |
|  | Production |  |  |  | 360 | 15 | 11 |  |  |  |
| 2016-17 | Indent |  |  |  | 340 | 60 | 30 |  |  |  |
|  | Production |  |  |  | 340 | 60 | 30 |  |  |  |
| 2017-18 | Indent |  |  |  | 340 | 130 | 20 |  |  |  |
|  | Production |  |  |  | 340 | 30 | 32 |  |  |  |
| 2018-19 | Indent |  |  |  | 640 |  | 40 | 60 |  |  |
|  | Production |  |  |  | 640 |  | 195 | 60 |  |  |
| 2019-20 | Indent |  |  |  | 240 | 10 | 10 | 150 | 10 |  |
|  | Production |  |  |  | 240 | 10 | 36 | 150 |  |  |
| 2020-21 | Indent |  |  |  | 250 |  | 32 |  | 75 | 75 |
|  | Production |  |  |  |  |  | 52 |  | 50 | 50 |
| 2021-22 | Indent |  |  |  | 175 |  |  | 20 | 100 | 15 |
|  | production |  |  |  | 175 |  |  | 20 | 23 | 15 |

Blank spaces represent no BS in demand or production or both in a particular year
